# Antimicrobial Activity and Chemical Composition of Essential Oil From the Seeds of Artemisia aucheri Boiss

**Published:** 2012-01-04

**Authors:** Gholamreza Asghari, Mohamad Jalali, Ehsan Sadoughi

**Affiliations:** 1 Department of Pharmacognosy, School of Pharmacy, Isfahan University of Medical Sciences, Isfahan, IR Iran; 2 School of Health, Food Security Research Center, Isfahan University of Medical Sciences, Isfahan, IR Iran; 3 Isfahan Pharmaceutical Research Center, Isfahan University of Medical Sciences, Isfahan, IR Iran

**Keywords:** Artemisia aucheri, Essential Oil, Antimicrobial Activity, Seed, Chemical Composition

## Abstract

**Background:**

Artemisia aerial parts are well known for antimicrobial activities including anti malaria.

**Objectives:**

This study was carried out to evaluate the antimicrobial activity and chemical composition of essential oil from the seeds of Artemisia aucheri Boiss (Asteraceae).

**Materials and Methods:**

Essential oil was extracted from the powdered seeds of Artemisia aucheri by hydrodistillation. Antimicrobial activity against five bacterial species was tested using the disc diffusion method, and the chemical composition of the essential oil was analyzed by gas chromatography-mass spectrometry (GC-MS).

**Results:**

The essential oil of Artemisia aucheri seed showed activity against Escherichia coli, Staphylococcus aureus, and Listeria monocytogenes. The essential oil constituents identified by GC-MS were as follows: decane, ρ-cymene, 1,8-cineole, linalool, ρ-mentha-8-ol, triene, borneol, lavandulol, bornyl acetate, chrysanthenyl acetate, dehydro aromadenderene, and caryophyllene oxide. Most of these compounds are also found in the aerial parts of Artemisia aucheri.

**Conclusions:**

Variation in the compositions of essential oils from Artemisia aucheri, and thus variation in the antimicrobial activity of these oils, may be due to the plant parts used for essential oil prepration.

## 1. Background

Since ancient times, herbs and their essential oils have been known to have varying degrees of antimicrobial activity (1). Natural plant resources that contain antimicrobial constituents have been extensively studied (2). Plants of the Asteraceae family are well known for their antimicrobial activity. The antimicrobial activity of Achillea spp. (3, 4), Arctotis auriculata Jacq., and Eriocephalus africanus L. (5, 6) have been reported. Further, there are also many reports on the bioactivities, including antimicrobial activity, of extracts and essential oils from species of the genus Artemisia (7). For instance, Artemisia douglassiana is used for its antifungal and antibacterial activities (8). This species has also been used as a topical bactericidal agent for skin burns (9). In addition, Artemisia annua is known for its antimalarial, antibacterial, antioxidant, and anticarcinogenic properties (10). The genus Artemisia comprises approximately 400 species, which are predominantly distributed in the northern temperate regions of the world (11). Among these species, 34 have been reported in Iran, some of which are endemic (12, 13). Various species of the genus Artemisia are used for their pharmacological, antimicrobial, and antioxidant activities (14-17). In traditional medicine, A. aucheri Boiss is a plant with astringent and disinfectant properties, and with antileishmanial, antiparasitic, and antioxidant activities (18). The major components of the oil obtained from the aerial parts of the plant have been reported (19-21).

## 2. Objectives

The chemical composition of the essential oil of A. aucheri seeds has not previously been published, nor has information on the antimicrobial activity of essential oil from the seeds of this plant. Therefore, in this study, the in vitro antibacterial properties and the chemical composition of the essential oil of A. aucheri seeds were investigated.

## 3. Materials and Methods

### 3.1. Plant Materials and Essential Oils

A. aucheri seed was purchased from Pakan Bazr Company, Isfahan, IR Iran. The seeds were ground to a coarse powder in a mill. The essential oil was obtained by hydro-distillation of 300 g of the powdered seed for 4 h using a Clevenger-type apparatus to collect the oil.

### 3.2. Gas Chromatography-Mass Spectrometry (GC-MS) Analysis 

The hydrodistilled seed oil of A. aucheri was analyzed by GC and GC-MS. Gas chromatographic analysis was carried out in a Perkin-Elmer 8500 gas chromatograph equipped with an FID detector and a BP-1 capillary column (39 m × 0.25 mm; film thickness 0.25 µm). The carrier gas was helium with a flow rate of 2 mL/min, and the oven temperature was maintained at 60°C for the first 4 min, and then increased at a rate of 4°C/min until reaching a temperature of 280°C. The injector and detector temperatures were set at 280°C. 

Confirmation of peak identity was accomplished by GC-MS. The mass spectra were recorded in an Agilent 7890 MS detector coupled with an Agilent 7890 gas chromatograph equipped with an HP-5MS capillary column (30 m × 0.25 mm; film thickness 0.25 µm). The GC conditions were as described above. The mass spectrometer conditions were as follows: ionized potential, 70 eV; source temperature, 200°C. Identification was based on retention times and computer matching with the WILEY 275.L library, and by comparison of electron impact mass spectra (EI-MS) with relevant reference samples and with previously reported data (22).

### 3.3. Microorganisms Used to Examine Antimicrobial Activity 

In vitro antimicrobial studies were carried out against five bacterial species: Escherichia coli PTCC 1338, Staphylococcus aureus PTCC 1337, Pseudomonas aeruginosa PTCC 1074, Salmonella enteritidis RITCC 1624, and Listeria monocytogenes RITCC 1293. 

### 3.4. Antimicrobial Activity 

20 μL of essential oil diluted with n-pentane were prepared as follows: 1:2, 1:4, 1:8 and 1:16. Antimicrobial tests were carried out using the disc diffusion method in Petri dishes (9 cm diameter) containing 10 ml of Mueller-Hinton Agar (Oxoid) inoculated with 1 mL of suspension containing 10^7^ CFU (colony-forming units)/mL of the target bacteria. Inocula were prepared by incubation of the bacteria in nutrient broth at 37°C for 24 h. One milliliter of each bacterial suspension was mixed with Mueller-Hinton agar and seeded onto the solidified Mueller-Hinton agar. The discs (6 mm diameter) were impregnated with 20 μL of extract or essential oil and then placed on the inoculated agar. The Petri dishes were maintained at 4°C for 2 h, after which the inoculated plates were incubated at 37°C at 24 h. Antimicrobial activity was determined by measuring the zones of inhibition against the test organisms (23).

## 4. Results

GC-MS analysis of the essential oil identified 11 main compounds. The results are presented in detail in Table 1. The oil consisted of alkanes, oxygenated and non-oxygenated monoterpenes, and sesquiterpenes. The oxygenated monoterpenes were either alcohols or esters, and the oxygenated sesquiterpenes were in the form of acetates and oxides. The results of the antimicrobial activity of the essential oil from A. aucheri seeds are presented in detail in Table 2. The oil was active against S. aureus, E. coli, and L. monocytogenes.

**Table 1:** The Main Components Present in the Seed Essential Oils of Artemisia aucheri Boiss

Constituents	%	RT ^a^	KI^b^	EI ^a, b^
Decane	5.4	5.08	1000	43, 57, 41, 71, 85
Para-Cymene	1.7	5.64	1025	119, 91, 134, 59, 117, 41
1,8-Cineol	3.3	5.81	1031	43, 81, 71, 69, 84
Linalool	27.1	7.62	1097	71, 93, 41, 69, 43, 80,154
Menthe-3-en, 8-ol	2.1	8.06	1150	41, 69, 68, 81, 55
Borneol	7.8	9.50	1169	95, 110, 41, 121, 139, 154
Lavandulol	4.1	9.56	1181	69, 41, 111, 68, 93
Chrysanthenyl acetate	2.3	12.47	1265	119, 43, 91, 113, 194
Bornyl acetate	2.7	13.23	1289	95, 93, 45, 121, 41
Dehydro-aromadendrene	2.3	21.96	14.63	41, 91, 77, 105, 161
Caryophyllene oxide	4.7	22.12	15.83	93, 79, 41, 91, 69

^a^Major fragments in order of decreasing m/z

^b^Abbreviations: EI, Electron Impact; KI, Kovat Index; RT, Retention Time

**Table 2:** Inhibition Zones of Artemisia aucheri Essential Oils Against Test Microorganisms Using the Disc Diffusion Method

Dilution Strength	100%, mean ± SD	50%, mean ± SD	25%, mean ± SD	12.5%, mean ± SD	Control ^a, b^0% (Neg.Control), mean ± SD
*S. aureus*	18.2 ± 1	14.3 ± 4	10.9 ± 1.7	7.9 ± 4	37 ± 1.15
*E. coli*	9.3 ± 0.58	5.5 ± 1.5	-	-	29 ± 1.2
*P. aeruginosa*	-	-	-	-	19.5 ± 0.5
*S. enteritidis*	-	-	-	-	25 ± 0.58
*L. monocytogenes*	13.5 ± 0.58	9 ± 1	-	-	32 ± 1.7

The data are expressed as the mean ± SD

^a^ciprofloxacin (positive control)

^b^n-pentane (negative control)

### 4.1. Phytochemical Analysis 

The results of the gas chromatographic analysis of A. aucheri oil are presented in Table 1. The constituents identified were as follows: decane, ρ-cymene, 1,8-cineol, linalool, ρ-mentha-8-ol, triene, borneol, lavandulol, and bornyl acetate as monoterpene constitiuents; and chrysanthenyl acetate, dehydro-aromadenderene, and caryophyllene oxide as sesquiterpene constituents. Decane (5.4%) was the only alkane identified in the oil. As clearly illustrated in Table 1, decane, as an unbranched alkane, gave rise to a significant homologous series of alkyl ions, C_n_H_2n+1_^+^, thereby showing a typical spectra that could be recognized on sight. The C_n_H_2n+1_^+^ ion alkyl series gives peaks at m/z 57, 71, 85, etc., and could be traced from C_4_H_9_^+^ to C_10_H_21_^+^. All of the important peaks except M^+^ were ions with an even number of electrons. The rates of initial decomposition of any molecular ion involving cleavages of different carbon-carbon bonds are comparable to each other, as are the rates of secondary decomposition of the primary product ions. This accounts for the regular increase in concentration with decreasing size of the alkyl ions. The possibility of rearranged products of greater stability becomes higher with the secondary reactions, so that the smaller ions, such as C_3_H_7_^+^ and C_4_H_9_^+^, are generally the more stable branched carbonium structures. Thus, the distribution of ions is maximized in the C_3_ and C_4_ region of the higher alkanes (24).

The hydrocarbon monoterpene ρ-cymene (1.7%) was also present in the oil. A characteristic of the spectra of most cyclic hydrocarbon monoterpenoids is the prominent M-34 ion m/z 93. ρ-Cymene has the most intense fragment ions at higher m/z-values (m/z 93 and above), indicating a certain stability of the ring system (25). ρ-Cymene, being an aromatic hydrocarbon, gave large fragment ions at m/z 134(M), m/z 119 (M-15)(base peak), and m/z 91(M-43). 

Linalool (27.1%) was the main alcohol monoterpene constituent of the oil. The other major monoterpene alcohol components were borneol (7.8%), lavandulol (4.1%), and 1,8-cineol (3.3%). The fragmentation spectra of the monoterpene alcohols, 1.8-cineol, linalool, borneol, and lavandulol, are shown in Table 1. The mass spectra of alcohols are generally more complex and less similar to one another owing to the influence of the polar hydroxyl group and its position in the structure. The spectra of the acyclic monoterpene alcohol linalool has the parent peak at m/z (M) 154 with m/z 71 as the base peak. The latter fragment is formed by splitting the bond that is in the allylic position relative to the double bond present in the respective molecules. The presence of a hydroxyl group is indicated by the occurrence of m/z (M-18) or of a fragment derived from this ion. The fragment m/z 93 is due to (M-18-43). As expected, the spectra of the analogue compounds lavandulol and linalool do not differ substantially. The mass spectra of the bicyclic monoterpene alcohol borneol is also shown in Table 1. The base peak for borneol is m/z 95 (M-18-15), and a second peak is at m/z 139(M-15). Caryophyllene oxide (4.7%) was the main sesquiterpene constituent, followed by chrysanthenyl acetate (2.3%) and dehydro-aromadendrene (2.3%). Aromadendrene, which is a simple sesquiterpene, may undergo fragmentation in a manner similar to the corresponding monoterpenoids (26). Thus, it will yield an intense peak at m/z 69 arising from the loss of the isopentenyl end group by allylic cleavage as described above. 

Most of these compound identified from A. aucheri seed oil have also been reported in other *Artemisia* species. However, earlier studies have also indicated that A. *scoparia* contains beta-pinene, A. *diffusa* contains camphor, and A. *turanica* contains 1,8-cineol as major constituents of their oils (27). Further, davanone has been identified as a major compound in the oil of A. *persica* (28). It is well known that the concentration of mono- and sesquiterpenoids in aromatic plants varies from species to species and with growth stage and seasonal variation (29-31).

The composition of the essential oil from the seeds of A. *aucheri* determined in the present study shows little similarity to that of the essential oil from the plant’s aerial parts investigated in previous studies (20). There has been controversy over the composition of these oils. Some authours have reported that the oil obtained from the aerial parts is mainly composed of geranyl acetate (17.2%), a-citral (17.1%), linalool (12.7%), geraniol (10.7%), and Z-citral (10.5%) (30). Another study has indicated that the major components are camphor (45.5%) and 1,8-cineole (14.3%) (32), whereas other researchers have found that the essential oil of A. aucheri is rich in linalool (44.1%), geranyl acetate (10.7%), (E)-citral (9.7 %), and (Z)-citral (7.7 %) (33). Similarly, camphor (22.87%) was determined to be the main component of the plant essential oil (34). Sefidkon et. al. have reported that the main constituents of the oil extracted from A. aucheri plants collected from Semnan Province, Iran, are verbenone (21.5%), camphor (21.0%), 1,8-cineol (8.3%), and trans-verbenol (8.1%) (35). Linalool is the only compound that has been found to occur in both the aerial parts and the seeds of the plant. These variations in the composition of the A. aucheri essential oils may due to variations in environmental parameters, such as irradiance, climate, nutrients, soil water availability, or to seasonal adaptations. It is well known that medicinal plant materials derived from the same species can show significant differences in quality when collected at different sites, owing to the influence of soil, climate, and other factors. These differences may also relate to physical appearance or to variations in their constituents, the biosynthesis of which may be affected by extrinsic environmental conditions, including ecological and geographical variables. 

### 4.2. Antimicrobial Activity

The antimicrobial effects of A. aucheri essential oils were tested against two gram-positive bacterial species (L. monocytogenes, S. aureus) and three gram-negative bacterial species (E. coli, P. aeruginosa, S. enteritidis). Corresponding solvents had no inhibitory effect on any of the test microorganisms in the control treatment. The results of these tests, as well as the effects of a control antibiotic, are presented in Table 2.

## 5. Discussion

As shown in Table 2, the essential oil of A. aucheri seeds showed some, albeit limited, antimicrobial activity against three of the five microorganisms tested: E. coli, S. aureus, and L. monocytogenes. These limited antimicrorbial effects may be due to the different composition of the essential oil of seeds compared with that of the oil obtained by hydrodistillation of aerial plant parts. The results of the present study indicate that extraction of the essential oil from the seeds instead of from the aerial parts resulted in the loss of several previously identified antimicrobial constituents of the oils. Terpene molecules such as geraniol and geranyl acetate, which are found in the essential oils of the aerial parts (36), and are reported to contribute to the antimicrobial activities, were not found in the seed extracts, whereas a number of other molecules, such as the sesquiterpenes, that were found in the seed oils (Table 1) have no antimicrobial activity. It has been reported that components of the essential oils obtained from the aerial parts of A. Aucheri plants showed antimicrobial activity against all tested microorganisms, including E. coli and Leishmania major (36, 37). When we compared the previously published results with those of the current study, it was apparent that the antimicrobial activity is strongly affected by the essential oil composition. It is well known that the concentration of biologically active constituents varies with the plant parts, which directly reflects this activity.
